# Protective effect of gallic acid against cisplatin-induced ototoxicity in rats^☆^

**DOI:** 10.1016/j.bjorl.2018.03.001

**Published:** 2018-04-07

**Authors:** Korhan Kilic, Muhammed Sedat Sakat, Fazile Nur Ekinci Akdemir, Serkan Yildirim, Yavuz Selim Saglam, Seda Askin

**Affiliations:** a Ataturk University, Faculty of Medicine, Department of Otorhinolaryngology, Erzurum, Turkey; b Agri Ibrahim Cecen University, High School of Health, Department of Nutrition and Dietetics, Agri, Turkey; c Ataturk University, Faculty of Veterinary, Department of Pathology, Erzurum, Turkey; d Ataturk University, Faculty of Medicine, Department of Biochemistry, Erzurum, Turkey

**Keywords:** Cisplatin-induced ototoxicity, Gallic acid, Oxidative stress, DPOAE, Ototoxicidade induzida por cisplatina, Ácido gálico, Estresse oxidativo, EOAPD

## Abstract

**Introduction:**

Cisplatin is an antineoplastic agent widely used in the treatment of a variety of cancers. Ototoxicity is one of the main side-effects restricting the use of cisplatin.

**Objective:**

The purpose of this study was to investigate the protective efficacy of gallic acid, in biochemical, functional and histopathological terms, against ototoxicity induced by cisplatin.

**Methods:**

Twenty-eight female Sprague Dawley rats were included. Rats were randomly assigned into four groups of seven animals each. Cisplatin group received a single intraperitoneal dose of 15 mg/kg cisplatin. Gallic acid group received intraperitoneal gallic acid at 100 mg/kg for five consecutive days. Cisplatin + gallic acid group received intraperitoneal gallic acid at 100 mg/kg for five consecutive days and a single intraperitoneal dose of 15 mg/kg cisplatin at 3rd day. A control group received 1 mL intraperitoneal saline solution for five consecutive days. Prior to drug administration, all rats were exposed to the distortion product otoacoustic emissions test. The test was repeated on the 6th day of the study. All rats were then sacrificed; the cochleas were removed and set aside for biochemical and histopathological analyses.

**Results:**

In cisplatin group, Day 6 signal noise ratio values were significantly lower than those of the other groups. Also, malondialdehyde levels in cochlear tissues were significantly higher, superoxide dismutase and glutathione peroxidase activities were significantly lower compared to the control group. Histopathologic evaluation revealed erosion in the stria vascularis, degeneration and edema in the connective tissue layer in endothelial cells, impairment of outer hair cells and a decrease in the number of these calls. In the cisplatin + gallic acid group, this biochemical, histopathological and functional changes were reversed.

**Conclusion:**

In the light of our findings, we think that gallic acid may have played a protective role against cisplatin-induced ototoxicity in rats, as indicated by the distortion product otoacoustic emissions test results, biochemical findings and immunohistochemical analyses.

## Resumo

**Introdução:**

A cisplatina é um agente antineoplásico amplamente usado no tratamento de vários tipos de câncer. A ototoxicidade é um dos principais efeitos colaterais que restringem o uso da cisplatina.

**Objetivo:**

O objetivo deste estudo foi investigar a eficácia protetora do ácido gálico, em termos bioquímicos, funcionais e histopatológicos, contra a ototoxicidade induzida por cisplatina.

**Método:**

Vinte e oito ratas Sprague-Dawley foram incluídas. As ratas foram distribuídas aleatoriamente em quatro grupos de sete animais cada. O grupo cisplatina recebeu uma única dose intraperitoneal de 15 mg/kg de cisplatina. O grupo ácido gálico recebeu ácido gálico via intraperitoneal a uma dose de 100 mg/kg durante cinco dias consecutivos. O grupo cisplatina + ácido gálico recebeu ácido gálico via intraperitoneal a uma dose de 100 mg/kg durante cinco dias consecutivos e uma única dose intraperitoneal de 15 mg/kg de cisplatina no terceiro dia. O grupo controle recebeu 1 mL de solução salina via intraperitoneal por cinco dias consecutivos. Antes da administração do fármaco, todos os ratos foram expostos ao teste de emissões otoacústicas - produto de distorção. O teste foi repetido no sexto dia do estudo. Todos os ratos foram então sacrificados; as cócleas foram removidas e reservadas para análises bioquímicas e histopatológicas.

**Resultados:**

No grupo cisplatina, os valores da relação sinal-ruído do dia 6 foram significativamente mais baixos aos dos outros grupos. Além disso, os níveis de malondialdeído nos tecidos cocleares foram significativamente mais altos, e as atividades de superóxido dismutase e glutatione peroxidase foram significativamente mais baixas em comparação com o grupo controle. A avaliação histopatológica revelou erosão na estria vascular, degeneração e edema na camada de tecido conjuntivo em células endoteliais, comprometimento das células ciliadas externas e diminuição do número dessas células. No grupo cisplatina + ácido gálico, estas alterações bioquímicas, histopatológicas e funcionais foram revertidas.

**Conclusão:**

Tendo em vista os nossos achados, consideramos que o ácido gálico pode ter desempenhado um papel protetor contra a ototoxicidade induzida por cisplatina em ratas, conforme indicado pelos resultados do teste emissões otoacústicas - produto de distorção, achados bioquímicos e análises imuno-histoquímicas.

## Introduction

Cis-diammineedichloroplatinum (cisplatin) is a chemotherapeutic agent widely used in the treatment of cancer since being approved by the Federal Drug Administration in 1978. The importance of long-term complications developing in association with chemotherapeutics is growing as survival rates in cancer patients improve. Side-effects such as ototoxicity, nephrotoxicity, and neurotoxicity are seen following cisplatin chemotherapy. Hearing losses at rates between 30% and 100% have been reported in association with cisplatin ototoxicity.^1^ This hearing loss results in multifaceted decreases in quality of life and can affect social and academic development, particularly in children.^2^

Cisplatin-induced ototoxicity involves high-frequency bilateral and symmetrical hearing loss. Such losses may be permanent and irreversible and are frequently associated with tinnitus.^3^ The severity of this hearing loss is associated with the frequency of drug application and the dosage concerned. No methods other than reducing the cisplatin dosage or modification to chemotherapeutic regimens not containing cisplatin have to date been discovered for the purpose of reducing cisplatin-related ototoxicity. These two methods may also have adverse impacts on treatment outcomes. Various studies have therefore investigated agents capable of preventing cisplatin-induced ototoxicity. Several antioxidant agents including d-methionine, allopurinol, flunarizine, curcumin and lutein have been employed for this purpose in experimental studies.^4^, ^5^, ^6^, ^7^, ^8^

Gallic acid is a trihydroxybenzoic acid, a phenolic acid found in numerous medicinal plants and food materials. It has long been known to exhibit powerful antioxidant effects.^9^ Phenolic acids have been reported to exhibit their antioxidant effects by directly scavenging free radicals, by inactivating enzymes responsible for Reactive Oxygen Species (ROS) production and increasing the activation of antioxidant enzymes.^10^

To the best of our knowledge, no previous studies have investigated the protective effect of gallic acid against cisplatin-induced ototoxicity. The purpose of this study was to investigate the protective efficacy of gallic acid in rats, in biochemical, functional and histopathological terms, against ototoxicity induced by cisplatin.

## Methods

### Animals

Approval for the study was granted by the Ataturk University Animal Studies Local Ethical Committee with number E.1700305147-11:145. Twenty-eight female Sprague Dawley rats weighing 220–240 g were included. The study was conducted in accordance with the Care and Use of Laboratory Animals Guide. All rats were housed in special cages with ad libitum access to chow and water in a stable temperature of 22° ± 1 °C and humidity of 45% ± 2%. The lighting system was adjusted to a 12 h light/12 h dark cycle.

### Experimental protocol

The gallic acid used in the study was obtained from the Sigma-Aldrich Chemical Company, and cisplatin from Kocak Pharma Co. (Kocak, Istanbul, Turkey). Rats were randomly assigned into four groups of seven animals each.

Group 1 (Cisplatin group): Received a single intraperitoneal dose of 15 mg/kg cisplatin.
Group 2 (Gallic acid group): Received intraperitoneal gallic acid at 100 mg/kg for five consecutive days.
Group 3 (Cisplatin + Gallic acid group): Received intraperitoneal gallic acid at 100 mg/kg for five consecutive days and a single intraperitoneal dose of 15 mg/kg cisplatin at 3rd day.
Group 4 (Control group): Received 1 mL intraperitoneal saline solution for five consecutive days.

Prior to drug administration, all rats underwent otoscopic examination under 40 mg/kg ketamine and 10 mg/kg xylazine anesthesia. All rats were then exposed to the Distortion Product Otoacoustic Emissions (DPOAE) test. The DPOAE test was repeated on the 6th day of the study under the same anesthesia protocol. All rats were then sacrificed, and the temporal bones were dissected. The cochleas were removed and right cochlea was set aside for biochemical analyses while the left cochlea was set aside for histopathological analyses.

### DPOAE procedure

DPOAE measurements was conducted with an Otometrics Madsen Capella device. This device was calibrated before each measurement. Before measurement, all rats were anesthetized with 40 mg/kg ketamine hydrochloride (Ketasol 10%, Richter Pharma Ag, Wels, Austria) and 10 mg/kg xylazine (Alfazyne 2%, Alfasan International BV, Voerden, the Netherlands). Following anesthesia, rats were subjected to otoscopic examination. Appropriate probes were placed on both right and left outer ear channels of the rats to perform DPOAE measurements in a silent room. Measurements were taken in the form of DPgrams. The difference between the levels of L1 and L2 was kept at 10 dB SPL (sound pressure level) (L1 = 65 dB SPL, L2 = 55 dB SPL). In order to obtain the most powerful response, the test was set to two separate frequencies, *f*1/*f*2 = 1.22. DPgram measurements were performed at frequencies of 2002, 2500, 3154, 4004, 5000, 6299, 7998 and 10,000 Hz. SNR values of 3 dB or above were regarded as positive. The Signal Noise Ratio (SNR) values of all groups obtained during the DPOAE test were compared within and between the groups.

### Histopathological analysis

Cochlear tissues collected for histopathological analysis were fixed for 48 h in 10% formalin solution. They were then softened for 96–120 h in Osteosoft (Merc, HC313331, Germany) decalcification solution and subsequently washed in running spring water. They were next processed in 80% alcohol (12 h × 2 times), 90% alcohol (12 h × 2 times), 96% alcohol (12 h × 2 times), 100% alcohol (12 h × 2 times), chloroform (5 h × 3 times), and liquid paraffin (12 h) before being embedded in paraffin blocks. Sections measuring 4 μm in thickness were taken from each block, and preparates were made ready on slides. The preparations readied for histopathological examination were then stained with Masson's trichrome and studied under a light microscope. Sections investigated under light microscopy were evaluated on the basis of lesions – none (−), slight (+), moderate (++) and severe (+++). Photographs were taken.

### Immunohistochemical examination

All sections placed onto adhesive slides (poly-l-Lysine) for immunoperoxidase examination were passed through xylol and alcohol series, deparaffinized and dehydrated. The procedure was performed according to the guideline of Ab80436 kit. Caspase-3 was used as primary antibody. Following the washing procedure, 3-3′ Diaminobenzidine (DAB) chromogen was dropped onto the section and allowed to stand for 5–10 min to absorb the chromogen. After emersion in Mayer's hematoxylin for 1–2 min for background staining, specimens were washed under tap water. The slides were then passed through alcohol and xylol series, covered, and examined under a light microscope (Leica DM 1000). Sections’ immune positivity was graded as none (−), slight (+), moderate (++) or severe (+++).

### Biochemical analysis

The cochleas set aside for biochemical examination were washed with physiological saline solution immediately after excision and then frozen at −80 °C. On the day of study, they were removed from the freezer, thawed, and homogenized with a 9 mL per gram 1.15% KCl solution. The homogenates were centrifuged at 10,000 rpm for 30 min for supernatant separation.

The spectrophotometric method described by Sun et al. was employed for Superoxide Dismutase (SOD) analysis.^11^ This technique is based on the reduction of Nitroblue Tetrazolium (NBT) by the superoxide producer xanthine–xanthine oxidase at 560 nm. SOD activity was expressed as U/L.

Malondialdehyde (MDA) analysis was performed using the Thiobarbituric Acid (TBA) technique described by Ohkawa and Yagi.^12^ This is based on spectrophotometric measurement of the pink product resulting from the reaction between MDA and TBA. The results were expressed as nmoL/mL.

Glutathione peroxidase (GPx) was measured using a modification of the method described by Paglia and Valentine.^13^ GPx is an enzyme that produces glutathione oxidation in a reaction in which hydrogen peroxide is reduced to water. Oxidized glutathione is again reduced to glutathione with the enzyme glutathione reductase in the presence of NADPH. The method is based on spectrophotometric measurement of the decrease in NADPH absorbance at 340 nm. GPx activity was expressed as IU/L.

### Statistical analysis

Statistical analysis was performed on SPSS 17.0 software. One-Way ANOVA was used for statistical analysis of normally distributed parameters, and the post hoc Tukey test was employed to identify sources of variation. The non-parametric Kruskal–Wallis test was used to analyze variations among data obtained using the semi-quantitative method at histopathological examination. Analyses between two groups were performed using the Mann–Whitney *U* test. *p* < 0.05 was regarded as statistically significant.

## Results

No significant difference was observed between the groups in terms of SNR values in the DPOAE results on the first day of the study. Also there was no difference between the right and left ear SNR values. When DPOAE results on Day 6 were compared with the findings from Day 1, no significant variation was observed in SNR values in Groups 2, 3 or 4. In Group 1, SNR values on Day 1 were significantly higher than those on Day 6 at all studied frequencies. This shows that cisplatin exerts an adverse effect on hearing functions by affecting the cochlea.

**Figure 1:**
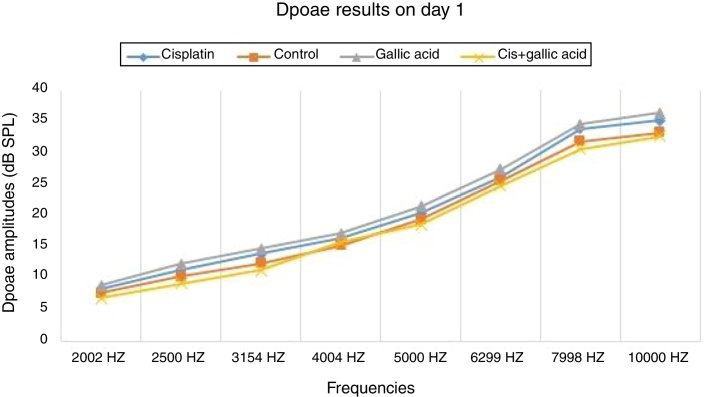
DPOAE results of groups on Day 1.

**Figure 2:**
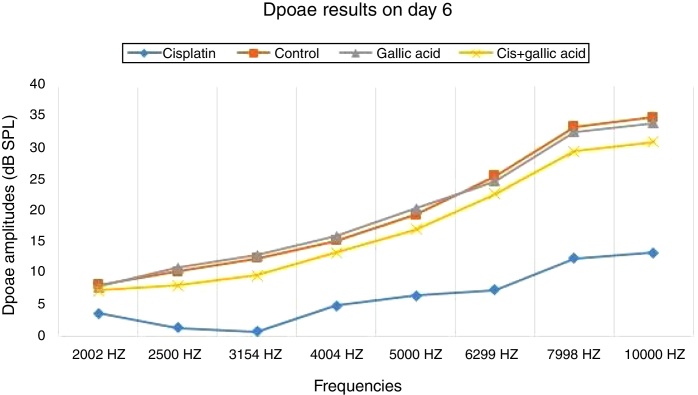
DPOAE results of groups on Day 6.

When the DPOAE test results on Day 6 were compared among themselves, SNR values in Group 1 were significantly lower than those of the other groups. No significant variation was determined between SNR values in Groups 2, 3 and 4. These findings show that the use of gallic acid with cisplatin prevented impairment in SNR values and exhibited functional protection against the ototoxic effect of cisplatin (Figure 1, Figure 2).

**Figure 3:**
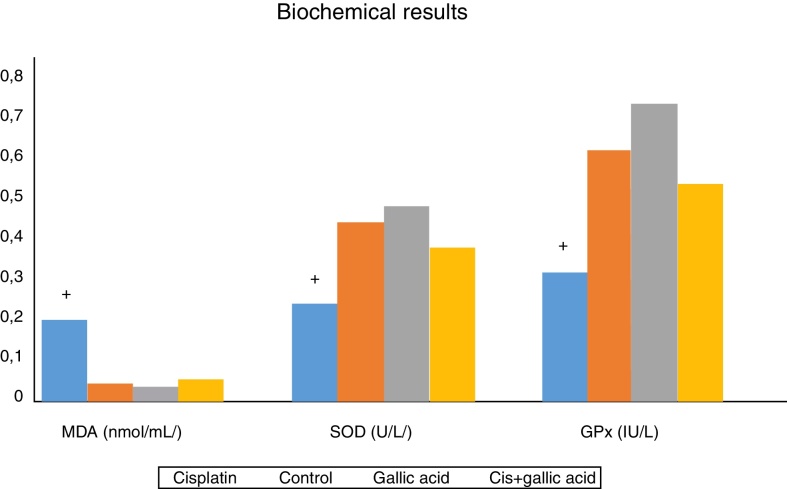
Biochemical results.

The results of biochemical analysis of rat cochleas are shown in Fig. 3. MDA levels in cochlear tissues in Group 1 were significantly higher compared to the control group. At the same time, SOD and GPx activities in cochlear tissues decreased significantly compared to the control group. This shows that oxidative stress occurred in the cochleas of rats receiving cisplatin, and that this stress was manifested with an increase in amounts of free oxygen radicals emerging with a rise in lipid peroxidation, and also with a decrease in antioxidant enzyme activities. No significant variation was observed between MDA levels, SOD and GPx activities in rats from Groups 2, 3 and 4. Additionally, MDA levels in the cochleas of rats from Group 3, those receiving gallic acid together with cisplatin, were significantly lower than those of rats in Group 1, those receiving cisplatin only, while SOD and GPx activities were significantly higher. This shows that gallic acid also protects against oxidative stress caused by the ototoxic effect of cisplatin.

**Figure 4:**
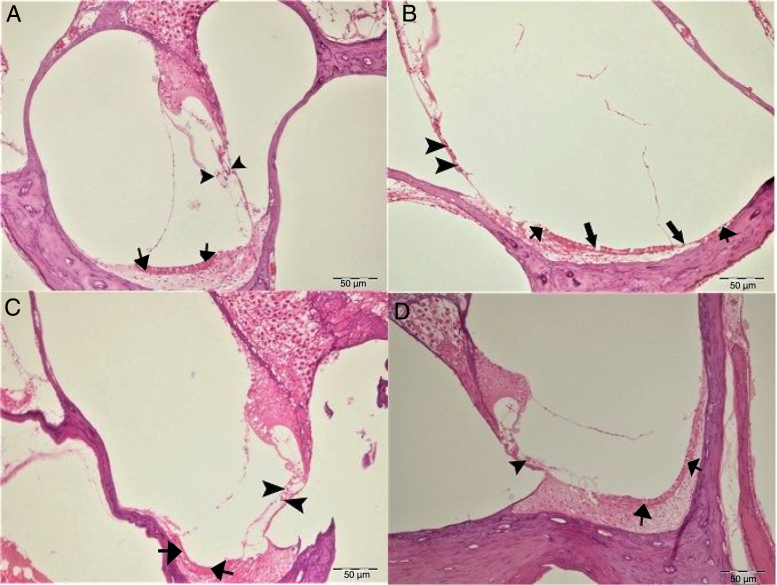
Histopathologic appearance of the cochlea, H&E, Bar: 50 μm. (A) Control Group, normal histopathological structure of the cochlea (arrow, stria vaskularis; arrow head, outer hair cells). (B) Cisplatin Group, hyperemia (thinner arrows), degeneration and erosion (thicker arrows) in the stria vascularis, decrease in the number of outer hair cells (arrow heads). (C) Gallic Acid Group, normal histopathological structure of the cochlea (arrow, stria vaskularis; arrow head, outer hair cells). (D) Cisplatin + Gallic Acid Group, mild hyperemia in the stria vaskularis (arrows), normal histopathological structure of outer hair cells with mild decrease in the number of these cells (arrow head).

In histopathologic examination, the cochleas in the control and gallic acid groups exhibited a normal histological appearance (Fig. 4A–C). Examination of the cochlea in the cisplatin group revealed erosion in the stria vascularis and degeneration and edema in the connective tissue layer in endothelial cells. The structure of outer hair cells was impaired, and a decrease in these cells was also observed (Fig. 4B). Examination of the cochleas in the Cis + gallic acid group revealed mild hyperemia in the stria vascularis, degeneration in epithelial cells and very mild degeneration in the spinal ganglia (Fig. 4D). These findings decreased in a statistically significant manner in the Cis + gallic group compared to the cisplatin group (*p* < 0.05). Histopathological cochlea findings are summarized in Table 1.

**Table 1:** Histopathologic findings of the cochlea.

	Hyperemia, degeneration and necrosis in stria vaskularis	Degeneration in spinal ganglia	Decrease in the number of outer hair cells	Immunopositivity of Caspase-3
Control group	−	−	−	−
Gallic acid group	−	−	−	−
Cisplatin group	+++	+++	+++	+++
Cis + Gallic acid	++	+	+	+

**Figure 5:**
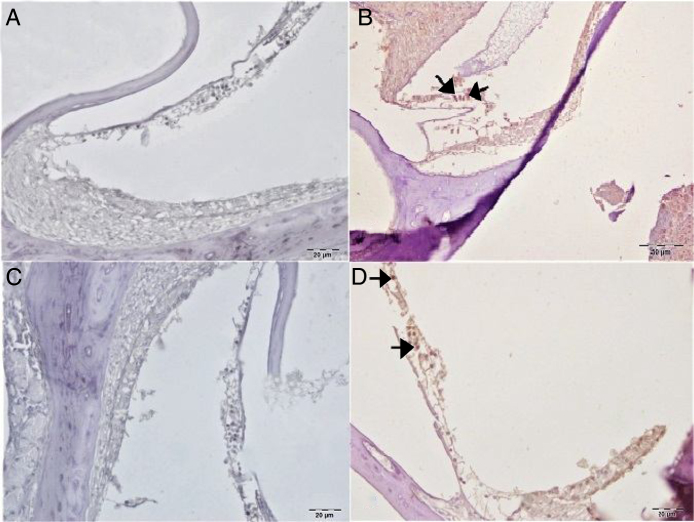
Immunohistochemical examination of the cochlea, IP, Bar: 5 μm. (A) Control Group, negative Caspase-3 expression in the outer hair cells. (B) Cisplatin Group, severe Caspase-3 immunopositivity in outer hair cells (arrow). (C) Gallic acid Group, negative Caspase-3 expression in the outer hair cells. (D) Cisplatin + Gallic acid Group, mild Caspase-3 immunopositivity in outer hair cells (arrow).

Caspase-3 staining was performed to determine apoptosis in cells. No Caspase-3 immunopositivity was observed at cochlear staining in the control or gallic acid groups (Fig. 5A–C). In the cisplatin group, severe intraplasmic immunopositivity was observed in outer hair cells (Fig. 5B). In the Cis + gallic acid group, mild intraplasmic Caspase-3 expression was determined in outer hair cells (Fig. 5D). A statistically significant decrease was determined compared to the cisplatin group (*p* < 0.05). Immunohistochemical findings are summarized in Table 1.

## Discussion

Our study clearly demonstrated the protective efficacy of gallic acid against cisplatin-induced ototoxicity. Gallic acid was determined, both biochemically and histopathologically, to prevent oxidative stress arising in the cochlea following cisplatin administration. The DPOAE findings also showed that gallic acid exerts a protective effect against hearing loss associated with cisplatin-induced ototoxicity.

Cisplatin is an antineoplastic agent widely used in the treatment of a variety of cancers. The main side-effects of this agent are nephrotoxicity, neurotoxicity and ototoxicity. While nephrotoxicity can be controlled with hydration therapy, ototoxicity remains the most significant side-effect of cisplatin use.^8^ The mechanism by which cisplatin leads to ototoxicity is not yet fully understood. Previous studies have shown that cisplatin causes hearing losses by impacting on various areas of the cochlea. The histopathological finding that has attracted the greatest interest is hair cell degeneration. Damage beginning in the tip links in the stereocilia of outer hair cells gradually leads to loss of all outer hair cells. This loss begins in the basal end and progresses to the apex. In addition, it causes collapse in Reissner's membrane and atrophy in support cells in the organ of Corti and in the stria vascularis.^14^, ^15^ In our study, histopathologic examination of the cochlea in the cisplatin group revealed erosion in the stria vascularis, degeneration and edema in the connective tissue layer in endothelial cells, impairment of the structure of outer hair cells and a decrease in the number of these cells.

The pathology responsible for this damage in the cochlea at the molecular and cellular level is not yet fully understood, although oxidative stress is believed to be the main agent. Cisplatin initiates numerous pathophysiological processes in the cochlea by increasing ROS production and suppressing antioxidant enzyme systems. Increased oxidative stress in the cochlea increases the peroxidation of lipid membranes, inactivates important cellular enzymes and compromises ion channel functions. Finally, increased ROS production leads to histopathological processes and functional impairments in the cochlea by causing apoptosis and necrotic cell death.^16^ In our study, Caspase-3 staining was performed to determine apoptosis in cells. Severe intraplasmic immunopositivity was observed in outer hair cells of the rats in the cisplatin group.

The cochlea has an effective antioxidant defense mechanism containing antioxidant such as vitamin C, vitamin E and Glutathione (GSH) and enzymes such as SOD, GPx and Catalase (CAT).^17^ SOD catalyzes the conversion of O_2_^−^ to H_2_O_2_ and O_2_, while CAT converts H_2_O_2_ to O_2_ and H_2_O. GPx reduces H_2_O_2_ and maybe certain other peroxides. Additionally, GPx also accelerates the conversion of reduced GSH to its oxidized form (GSSG) during the detoxification of H2O2.^16^ If this ROS elimination system is compromised, ROS lead to lipid peroxidation and cellular damage. This manifests itself with elevated MDA levels. Levels of MDA were significantly higher in the cochleas of rats receiving cisplatin compared to the control group in our study. However, SOD and GPx activities in the cochlear tissues of rats receiving cisplatin were significantly lower than those of the control group. These findings reveal that oxidative stress is involved in cochlear damage occurring in cisplatin-induced ototoxicity.

In the light of these physiological processes, it has been hypothesized that reducing ROS levels and increasing levels of antioxidant enzymes can be protective against cisplatin-induced ototoxicity. Various antioxidant agents have been investigated in cisplatin ototoxicity. The purpose of our study was to determine whether gallic acid, a powerful antioxidant, would exhibit a protective effect against cisplatin-induced ototoxicity.

Gallic acid is a poly-hydroxyphenolic compound found in numerous plants, fruits and foodstuffs. It also occurs naturally in a number of terrestrial and aquatic plants.^18^ It exhibits powerful anti-carcinogenic, anti-mutagenic and anti-inflammatory effects.^19^ Gallic acid and the derivatives thereof are powerful antioxidants capable of scavenging free radicals, such as superoxide anions, hydrogen peroxide (H_2_O_2_), hydroxyl radicals and hypochlorous acid.^19^ It has been suggested that this free radical scavenging activity may account for the ameliorative effect of gallic acid on oxidative stress.

Oyagbemi et al. showed that gallic acid ameliorates oxidative stress by suppressing ROS production in the rat brain and improving antioxidant status.^20^ Prince et al. reported that gallic acid increased the activity of SOD, CAT and GSH dependent enzymes in diabetic brain damage induced with streptozocin and prevented the formation of free radicals.^21^ Omobowale et al. also reported that gallic acid exhibited a protective effect against hepatic damage in doxorubicin-induced hepatotoxicity.^22^ Li et al. investigated the antioxidant substances that best prevented oxidative stress experimentally induced in ovarian tissue. They concluded that gallic acid functioned as a powerful antioxidant by reducing ROS production and by activating the antioxidant enzyme system.^23^ It was suggested in our study that, administration of gallic acid together with cisplatin showed a significant biochemical and histopathologic protection against cisplatin induced ototoxicity by acting as a powerful antioxidant.

The DPOAE test is an objective method that elicits functional outcomes of damage occurring in the cochlea in a rapid, inexpensive and non-invasive manner. Based on our DPOAE responses, significant impairment was observed in SNR values at high frequencies in rats receiving cisplatin compared to those in the control group. In addition, we documented significantly higher SNR values in rats administered gallic acid together with cisplatin compared to the cisplatin only group. This shows that gallic acid also exhibits a functional protective effect against ototoxicity induced with cisplatin.

## Conclusion

In the light of our findings, we think that gallic acid may have played a protective role against cisplatin-induced ototoxicity in rats, as indicated by the DPOAE results, biochemical findings and immunohistochemical analyses. Although our results do indicate that gallic acid can reduce cisplatin-induced ototoxicity in rats, its usefulness in the clinical setting remains uncertain. Further studies are now needed to determine the appropriate indications and dosages of gallic acid before clinical use against cisplatin-induced ototoxicity can be recommended.

## Conflicts of interest

The authors declare no conflicts of interest.
